# Identification of a six-gene signature to predict survival and immunotherapy effectiveness of gastric cancer

**DOI:** 10.3389/fonc.2023.1210994

**Published:** 2023-06-19

**Authors:** Qi Wang, Biyuan Zhang, Haiji Wang, Mingming Hu, Hui Feng, Wen Gao, Haijun Lu, Ye Tan, Yinying Dong, Mingjin Xu, Tianhui Guo, Xiaomeng Ji

**Affiliations:** Department of Radiation Oncology, The Affiliated Hospital of Qingdao University, Qingdao, Shandong, China

**Keywords:** gastric carcinoma, prognostic model, microenvironment, nomogram, bioinformatics

## Abstract

**Background:**

Gastric cancer (GC) ranks as the fifth most prevalent malignancy and the second leading cause of oncologic mortality globally. Despite staging guidelines and standard treatment protocols, significant heterogeneity exists in patient survival and response to therapy for GC. Thus, an increasing number of research have examined prognostic models recently for screening high-risk GC patients.

**Methods:**

We studied DEGs between GC tissues and adjacent non-tumor tissues in GEO and TCGA datasets. Then the candidate DEGs were further screened in TCGA cohort through univariate Cox regression analyses. Following this, LASSO regression was utilized to generate prognostic model of DEGs. We used the ROC curve, Kaplan-Meier curve, and risk score plot to evaluate the signature’s performance and prognostic power. ESTIMATE, xCell, and TIDE algorithm were used to explore the relationship between the risk score and immune landscape relationship. As a final step, nomogram was developed in this study, utilizing both clinical characteristics and a prognostic model.

**Results:**

There were 3211 DEGs in TCGA, 2371 DEGs in GSE54129, 627 DEGs in GSE66229, and 329 DEGs in GSE64951 selected as candidate genes and intersected with to obtain DEGs. In total, the 208 DEGs were further screened in TCGA cohort through univariate Cox regression analyses. Following this, LASSO regression was utilized to generate prognostic model of 6 DEGs. External validation showed favorable predictive efficacy. We studied interaction between risk models, immunoscores, and immune cell infiltrate based on six-gene signature. The high-risk group exhibited significantly elevated ESTIMATE score, immunescore, and stromal score relative to low-risk group. The proportions of CD4^+^ memory T cells, CD8^+^ naive T cells, common lymphoid progenitor, plasmacytoid dentritic cell, gamma delta T cell, and B cell plasma were significantly enriched in low-risk group. According to TIDE, the TIDE scores, exclusion scores and dysfunction scores for low-risk group were lower than those for high-risk group. As a final step, nomogram was developed in this study, utilizing both clinical characteristics and a prognostic model.

**Conclusion:**

In conclusion, we discovered a 6 gene signature to forecast GC patients’ OS. This risk signature proves to be a valuable clinical predictive tool for guiding clinical practice.

## 1 Introduction

Gastric cancer (GC) ranks as the fifth most prevalent malignancy and the second leading cause of oncologic mortality globally (1). There are several different types of GC, of which gastric adenocarcinoma accounts for 90% of total cases. The tumor, node, and metastasis (TNM) classification system and histological types are the most common methods for evaluating prognosis judgement and therapy guidance. Despite staging guidelines and standard treatment protocols, significant heterogeneity exists in patient survival and response to therapy for GC (2). Thus, an increasing number of research have examined prognostic models recently for screening high-risk GC patients. A large body of evidence suggests that immune cell infiltration in cancer has a critical function in carcinogenesis and progression, with much emphasis on predictive efficacy of immunotherapy (3, 4). A genetic analysis of Cancer Genome Atlas (TCGA) identified four distinct molecular subgroups of GC: Epstein-Barr virus (EBV) positive, microsatellite instability, genomic stability, and chromosomal instability (5). The EBV positive subtype presents with abundant PD-L1 expression, and has intensively described as a subset possibly profiting from immunotherapy (5). Infection with EBV triggers immune responses, and alters immune-related molecular components with immune cells recruitment (6). Although EBV positive GC patients are potentially eligible for immunotherapy theoretically, the efficacy of immune checkpoint inhibitors (ICIs) has been equivocal (7, 8). PD-L1 is a widely utilized prognostic biomarker for immunotherapy in variety of malignancies (9). Nevertheless, only around 20% of GC patients benefit from immunotherapy, and the immunological processes implicated in the processes are yet unknown (10). Due to high GC tumor heterogeneity, immune therapy can differ greatly from patient to patient. Furthermore, ICIs’ high cost and limited availability significantly restrict their clinical application. Thus, the need to learn more about GC pathogenesis heterogeneity and to find new immunotherapeutic targets and prognostic markers has attracted increasing attention in recent years.

The current investigation utilized the Gene Expression Omnibus (GEO) and TCGA databases to formulate a prognostic signature of six genes for GC patients. At first, we explored differentially-expressed genes (DEGs) from TCGA and GEO databases across GC and adjacent non-tumor tissues. The gene ontology (GO) enrichment analysis and Kyoto Encyclopedia of Genes and Genomes (KEGG) indicated DEGs were potentially involved in modulation of tumor immune microenvironment (TME). We further screened survival-related signatures and constructed a six-gene prognostic model among TCGA dataset. Based on the six-gene signature, we examined the link between risk models, immunoscores, immune cell infiltration, and cancer cell stemness. Overall, our study explored a six-gene risk model that can potentially identify GC patient’s risk and predict immunotherapy response.

## 2 Methods and materials

### 2.1 Data source

Based on TCGA (https://portal.gdc.cancer.gov/), 375 GC samples and 32 non-tumor samples were downloaded, along with their mRNA expression profiles. Three independent datasets were obtained from GEO: GSE54129, GEO: GSE66229 (11), and GEO: GSE64951 (12). We obtained external validation data from GEO: GSE62254 (13). We examined the relationship across prognostic model and immunotherapy response in four immunotherapeutic cohorts: the IMvigor210 cohort (atezolizumab for locally advanced or metastatic urothelial cancer), the GSE78220 (melanoma with anti-PD-1 treatment), the GSE35640 (melanoma with MAGE-A3 immunotherapy), and GSE67501 (renal cell carcinoma with anti-PD-1 immunotherapy). The “IMvigor210CoreBiologies” R packages were utilized to retrieve the transcriptomic and clinical IMvigor210 variables. A log2 transformation was conducted using limma Bioconductor package to transform the expression data from each database into fragments per kilobase of transcripts per million mapped reads (FPKMs). Gene symbols identified by multiple probes were computed based on their average expression levels.

### 2.2 Identification ferroptosis-related DEGs

By using limma Bioconductor package, DEGs between GC and adjacent non-tumor tissues were detected among TCGA dataset, GSE54129, GSE62259, and GSE64951. The threshold values in the GSE54129, GSE66229, and GSE64951 were as follows: log2|fold change| ≥1 and P-value<0.05. The generation of heatmap and volcano plot was performed through the utilization of R “pheatmap” package (14). We analyzed GO and KEGG enrichment analyses to assess possible DEG functions (15, 16). BiNGO plugin for Cytoscape was used to analyze GO enrichment in DEGs.

### 2.3 Prognostic model construction and validation

Utilizing R “survival” package, a univariate Cox regression was done on TCGA cohort to assess overall survival (OS) related genes. Subsequently, we proceeded to generate a prognostic signature by means of the least absolute shrinkage and selection operator (LASSO) Cox regression method utilizing R “glmnet” package (17) in TCGA cohort. Each GC patient’s risk score was determined utilizing the following:


$$\text{Risk score}=\sum_{i}^{n}\text{coefficient}\times \text{DEGs expression}\;$$

The GC patient cohorts were segregated into low-risk and high-risk groups by means of the risk score median value. Subsequently, the assessment and comparison of OS times across groups was performed through Kaplan-Meier plot. Furthermore, prognostic model was evaluated for its sensitivity and specificity utilizing R “time ROC” package (18).

### 2.4 Immune landscape-risk score relationship

Through gene expression data transformation, ESTIMATE is capable of identifying the purity and activity of stromal and immune cells within TME. R packages “estimate” (19) was utilized to compare immunescores abundance in high-risk and low-risk GC patients. By analyzing bulk samples using RNAseq profiles, the xCell can estimate 64 immune cell types abundance. R “xCell” packages were utilized to analyze xCell scores (20). Each GC patient’s tumor-infiltrating immune cells and risk score were calculated relative to their abundance.

A total of eight transcripts were chosen for analysis based on their relevance to immunological checkpoints; as CD274, CTLA4, HAVCR2, LAG3, PDCD1, PDCD1LG2, TIGIT, and SIGLEC15. A heatmap illustrating risk scores and immune-checkpoint-relevant genes was generated utilizing R “pheatmap” package. We estimated potentiality of immunotherapy response utilizing TIDE algorithm (21).

### 2.5 Nomogram development and validation

To guide clinical decision-making, we developed a predictive nomogram combining predictive model risk score and clinical characteristics. A P-value<0.05 was used to screen survival-related clinical variables by univariate Cox analysis. After that, a nomogram was created using multivariate survival analysis. Calibration curves were utilized to plot nomogram predictions against measured rates. ROC curves were utilized to evaluate prognostic model’s specificity and sensitivity. We used R packages “rms”, “rmda”, and “time ROC” to plot the nomogram, calibration curve, and ROC curves.

### 2.6 Statistical analyses

The statistical analyses were done utilizing R Studio (V. 1.4). Students’ t-tests were used to determine the difference across normal and tumor samples. The spherical or Fisher’s tests were utilized when appropriate to determine if a correlation between risk score and clinical parameters existed. The Kaplan-Meier plot was utilized to assess survival time. All P values were 2-tailed at 0.05 significance level.

## 3 Results

### 3.1 Identifying DEGs that are related to a worse prognosis

**Figure 1:**
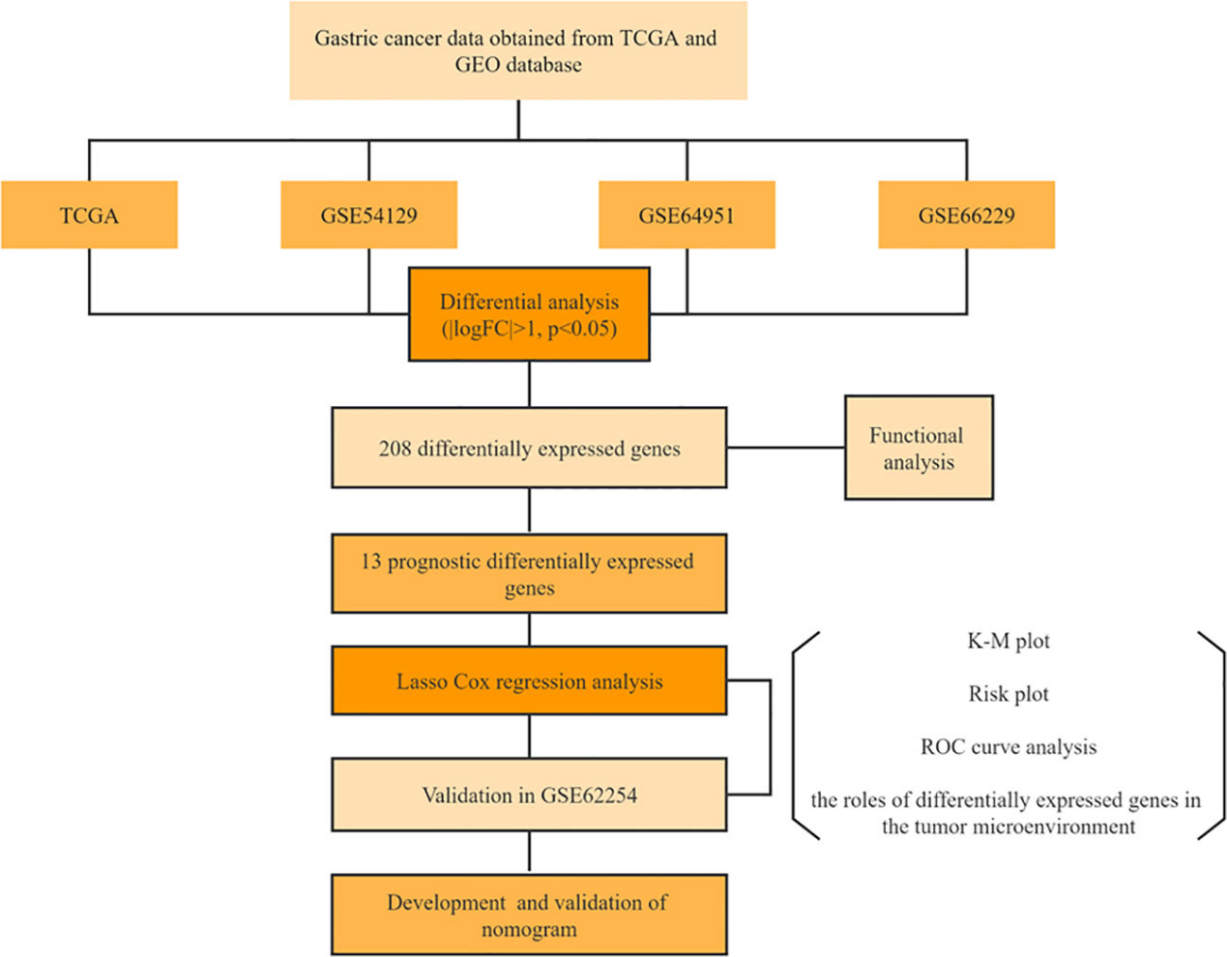
Flow chart.

**Figure 2:**
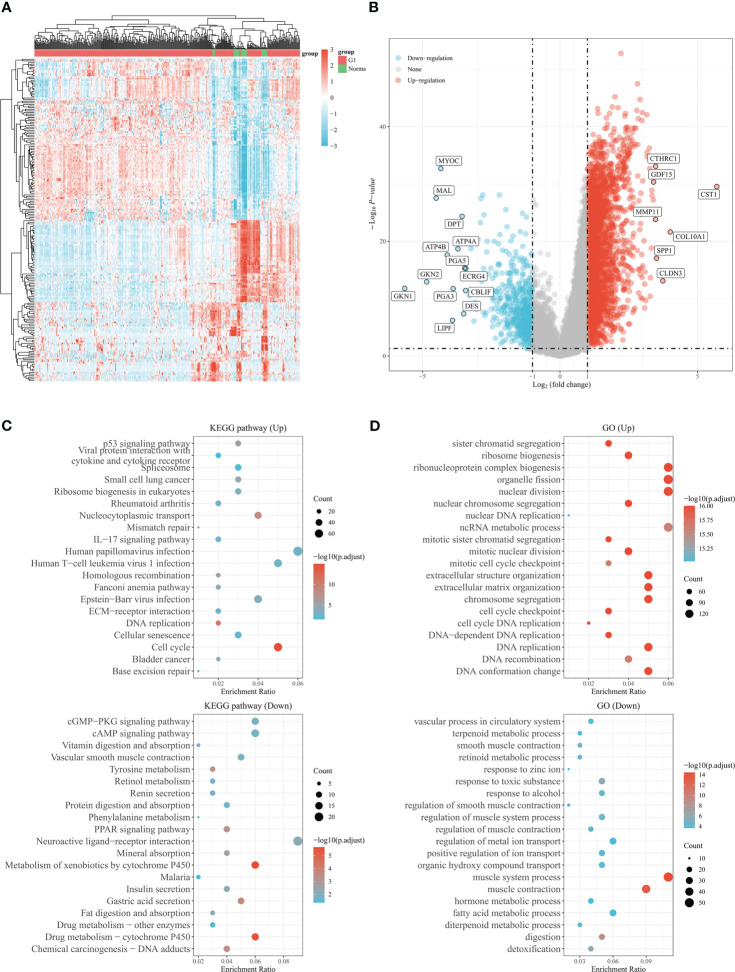
Genes differentially expressed in gastric cancer based on the TCGA database. **(A)** The heatmap shows differentially expressed genes in gastric cancer based on the TCGA database. **(B)** Volcano plot of differentially expressed genes in gastric cancer from TCGA database. Up- and down-regulated genes are indicated in red and blue, respectively. **(C)** Bubble graph for KEGG pathways. **(D)** Bubble graph for GO pathways.

The study’s procedure flowchart is depicted in **Figure 1**. From TCGA cohort, we obtained 3211 DEGs between GC tissues and adjacent non-tumor tissues. Among these, 2700 genes were upregulated, while 511 genes were downregulated. The heatmap and the volcano plot of DEGs are depicted in **Figures 2A, B**. Genes involved in mismatch repair, IL-17 signaling pathway, cell cycle, and base excision repair, were primarily upregulated. Pathways including cAMP signaling, protein digestion and absorption, PPAR signaling, Gastric acid secretion, and chemical carcinogenesis were highly enriched in downregulated genes (**Figure 2C**). In the GO functional analysis, the upregulated DEGs were chiefly enriched in nuclear division, mitotic cell cycle checkpoint, and DNA replication. In response to zinc ion, alcohol, and positive regulation of ion transport, the downregulated DEGs were enriched (shown in **Figure 2D**).

**Figure 3:**
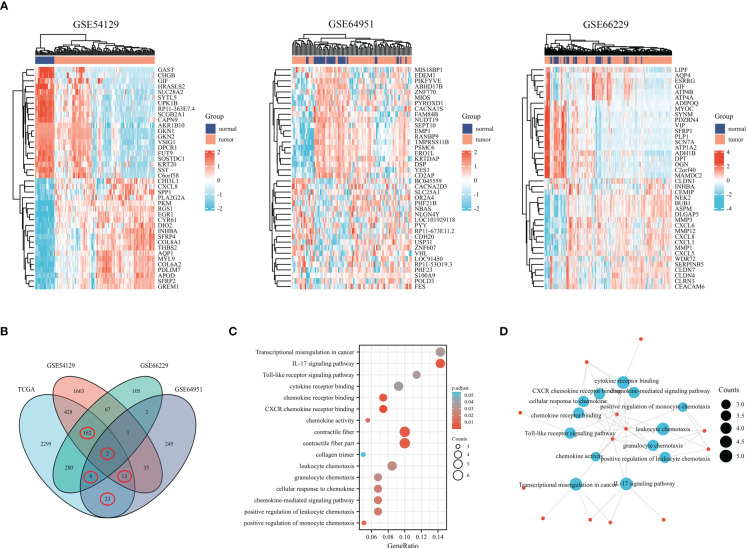
Genes differentially expressed in gastric cancer based on the GEO database. **(A)** The heatmap shows differentially expressed genes in gastric cancer based on the GSE54129, GSE64951, and GSE66229. **(B)** Venn diagram of differentially expressed genes. **(C)** Bubble graph of molecular function and biological process. **(D)** DEGs were enriched by GO biological terms using Cytoscape’s BiNGO plugin.

Under the cut-off threshold, there were 2371 genes in GSE54129, 627 genes in GSE66229, and 329 genes in GSE64951 were selected as prospective genes and intersected with to obtain DEGs (shown in **Figure 3A**). In total, the 208 DEGs were further screened in TCGA dataset through univariate Cox regression analysis (shown in **Figure 3B**). Functional enrichment analysis was conducted to understand DEGs underlying mechanisms among GC. The functional analyses revealed that DEGs were largely enriched across IL-17 signaling pathways, cytokine receptor binding, chemokine activity, cellular response to chemokine and positive regulation of leukocyte chemotaxis (shown in **Figures 3C, D**). We included 353 comprehensive clinical data samples among TCGA cohort for subsequent analysis and 13 genes were identified as predictive genes.

### 3.2 Prognostic model construction and validation

**Figure 4:**
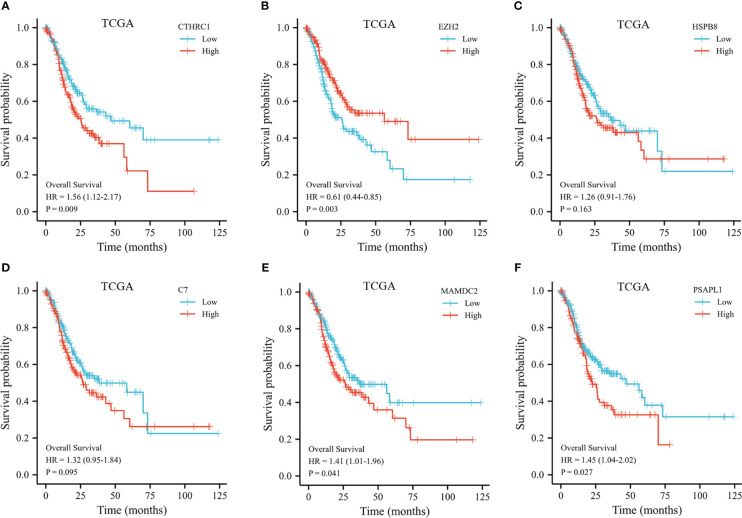
Kaplan-Meier plot of the selected genes from the TCGA dataset. **(A)** Kaplan-Meier plot of CTHRC1. **(B)** Kaplan-Meier plot of EZH2. **(C)** Kaplan-Meier plot of HSPB8. **(D)** Kaplan-Meier plot of C7. **(E)** Kaplan-Meier plot of MAMDC2. **(F)** Kaplan-Meier plot of PSAPL1.

**Figure 5:**
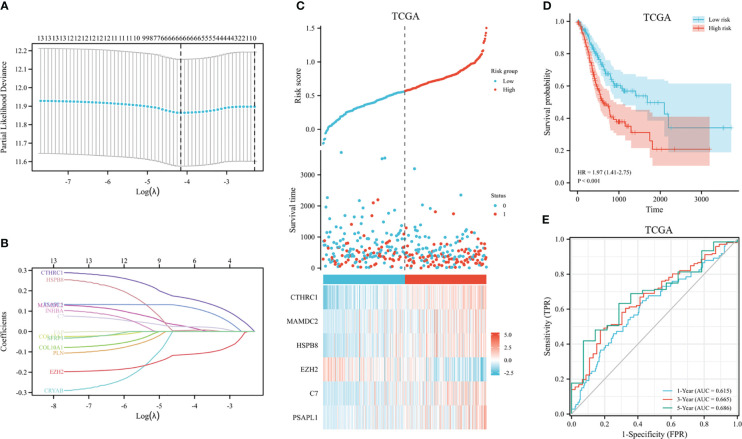
Construction of GC prognostic signature in the TCGA dataset. **(A)** The selection of optimal predictive variables by 10-fold cross-validation. **(B)** LASSO coefficients. **(C)** The risk plot between the high-risk and low-risk groups. **(D)** Analysis of overall survival between high-risk and low-risk groups. **(E)** The receiver operating curve for overall survival over time.

**Figure 6:**
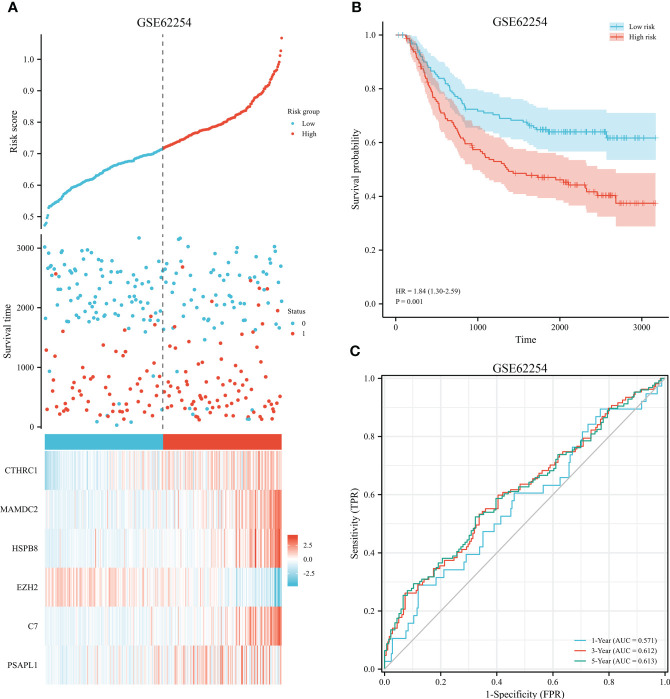
Validation of prognostic signature for GC. **(A)** Risk plot between the high-risk and low-risk groups in the external validation dataset. **(B)** High-risk versus low-risk survival analysis in the GSE62254. **(C)** Overall survival prediction curve based on GEO validation data.

The 353 GC patients from TCGA database were utilized as a training set. In order to verify prognostic signature accuracy and reliability, GEO GSE62254 was utilized as a validation cohort. Our study utilized TCGA dataset to establish a prognostic signature utilizing LASSO regression. The above 13 genes were further narrowed to 6 genes, namely, CTHRC1, MAMDC2, HSPB8, EZH2, C7, and PSAPL1. **Figures 4A–F** shows the Kaplan-Meier plots for these 6 genes. Based on LASSO regression analysis, prognostic signature was developed (shown in **Figures 5A, B**). Accordingly, risk scores were determined for each patient: 0.19×CTHRC1 + 0.06×MAMDC2 + 0.0005×HSPB8-0.13×EZH2 + 0.03*C7 + 0.15*PSAPL1. Based on validation and training sets, a risk score has been assigned to each patient. Patients were classified into high-risk and low-risk categories according to median risk score. **Figures 5C, D** demonstrates that high-risk patients had a lower OS rate than low-risk patients. **Figure 5E** shows that the prognostic signature was well established at 1-, 3-, and 5-year AUCs of 0.62, 0.67, and 0.69. Further validation of the proposed 6-gene prognostic model was conducted. As shown in **Figures 6A, B**, the prognostic model could determine the level of risk for GC patients based on Kaplan–Meier survival plot of validation set. Survival times were significantly shorter for high-risk scores patients than for those with low-risk scores (HR 1.84, 95% CI 1.30-2.59, *p<*0.01). In the validation set, the 1-, 3- and 5-year AUC values for risk score model were 0.57, 0.61 and 0.61, respectively (**Figure 6C**).

**Figure 7:**
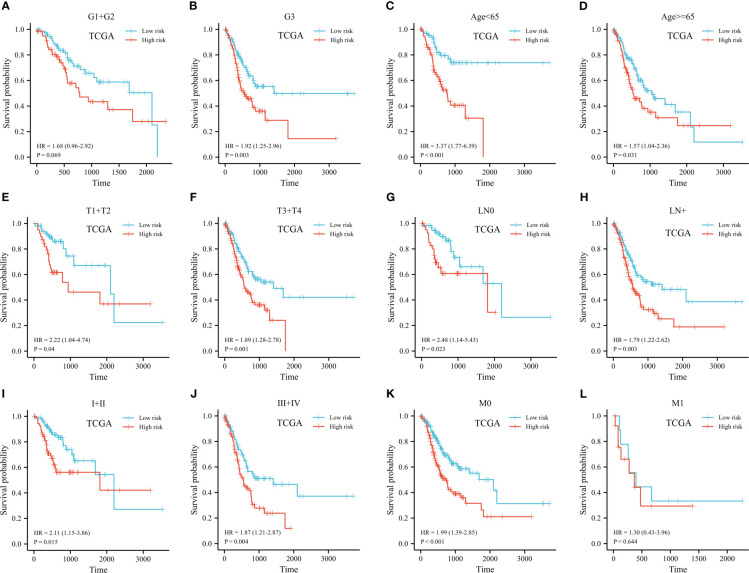
Kaplan-Meier plot of stratified analyses of the prognostic signature for associations with clinical characteristics. **(A)** OS plot in G1+G2 patients. **(B)** OS plot in G3 patients. **(C)** OS survival plot in patients older than 65 year-old. **(D)** OS survival plot in patients younger than 65 year-old. **(E)** OS survival plot in T1+T2 stage. **(F)** OS survival plot in T3+T4 stage. **(G)** OS survival plot in N0 stage. **(H)** OS survival plot in N+ stage. **(I)** OS survival plot in stage I+stage II. **(J)** OS survival plot in stage III+stage IV. **(K)** OS survival plot in M0 stage. **(L)** OS survival plot in M1 stage. (OS, overall survival; G, grade; T, tumor; N, lymph node; M, metastasis).

The predictive signature was stratified based on histopathological grade, age, and TNM stage. Low-histologic grade patients with high-risk groups had a HR of 1.68 (95% CI 0.96-2.92) (*p*=0.07, shown in **Figure 7A**). Despite this, prognostic risk model correctly identified short- and long-term survival groups for high-grade GC patients (HR 1.92, 95% CI 1.25-2.96, p=0.003, shown in **Figure 7B**). We further conducted stratification analysis based on age, T, N, and clinical staging. There was a significant correlation of risk scores with survival was found in both groups (shown in **Figures 7C-J**). Among patients with late M stage disease, the relationship was not noteworthy (HR 1.30, 95% CI 0.43-3.96, *p*=0.64, shown in **Figures 7K, L**).

### 3.3 Risk score-immune landscape relationship

**Figure 8:**
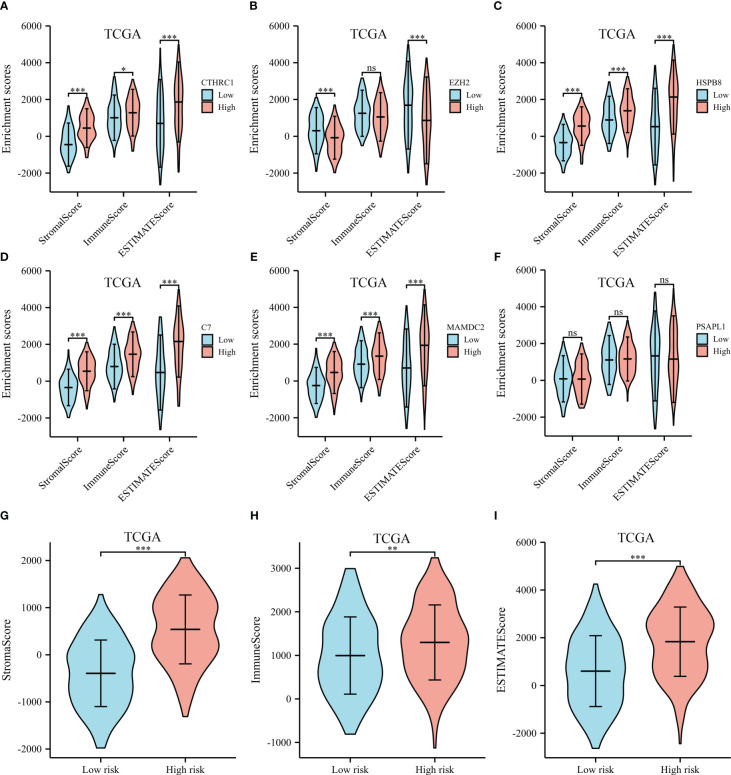
ESTIMATE scores of the selected genes from the TCGA dataset. **(A)** ESTIMATE scores of CTHRC1. **(B)** ESTIMATE scores of EZH2. **(C)** ESTIMATE scores of HSPB8. **(D)** ESTIMATE scores of C7. **(E)** ESTIMATE scores of MAMDC2. **(F)** ESTIMATE scores of PSAPL1. **(G)** Stromal score between the high- and low-risk groups. **(H)** Immune score between the high- and low-risk groups. **(I)** ESTIMATE score between the high- and low-risk groups. *p < .05, **p < .01, ***p < .001, and ns means not significant.

ESTIMATE, immune, and stromal scores were compared between the two groups to investigate possible biological mechanisms. The high expression of CTHRC1, MAMDC2, HSPB8, C7 and PSAPL1 was correlated with higher ESTIMATE, stromal and immune score, than low expression group (shown in **Figures 8A, C–F**). The opposite results were observed in EZH2 (**Figure 8B**). Moreover, our results showed high-risk patients had significantly higher immune, ESTIMATE, and stromal scores than low-risk (**Figures 8G–I**).

**Figure 9:**
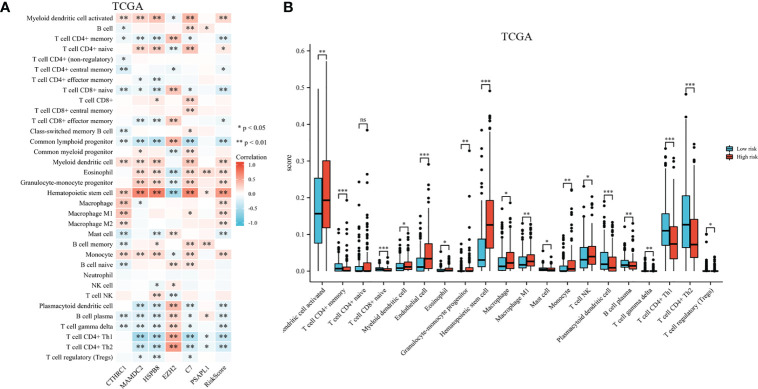
Infiltration of immune cells among high-risk groups versus low-risk groups. **(A)** Proportional heatmap of immune cells. **(B)** Bar graph illustrating differences in infiltrated immune cells in tumor microenvironments. *p < .05, **p < .01, ***p < .001, and ns means not significant.

A heatmap shows immune cell infiltrate across TCGA cohort groups (shown in **Figure 9A**). EZH2 expression had a significant positive relationship with CD4^+^ memory T cell, CD8^+^ naive T cell, CD8^+^ effector memory T cell, common lymphoid progenitor, mast cell, gamma delta T cell, CD4^+^ Th1 and Th2 T cell, and a negative relationship with common myeloid progenitor, eosinophil, hematopoietic stem cell, and T cell NK. Research has shown significant differences in CTHRC1, MAMDC2, HSPB8, and C7 expression levels among the activated myeloid dendritic cell, CD4^+^ memory T cell, CD8^+^ naive T cell, hematopoietic stem cell, monocyte, gamma delta T cell, and B cell plasma. As shown in **Figure 9B**, the proportions of activated myeloid dendritic cell, CD4^+^ memory T cell, myeloid dendritic cell, eosinophil, granulocyte-monocyte progenitor, hematopoietic stem cell, macrophage, macrophage M1, and monocyte were significantly elevated among high-risk. We also noted a significant rise in relative fractions of CD4+ memory T cells, CD8+ naive T cells, common lymphoid progenitor, mast cell, plasmacytoid dendritic cell, regulatory T cells, gamma delta T cell, and B cell plasma among low-risk group.

### 3.4 Survival-related gene signature related to immune-checkpoint–relevant genes and immunotherapeutic response among GC patients

**Figure 10:**
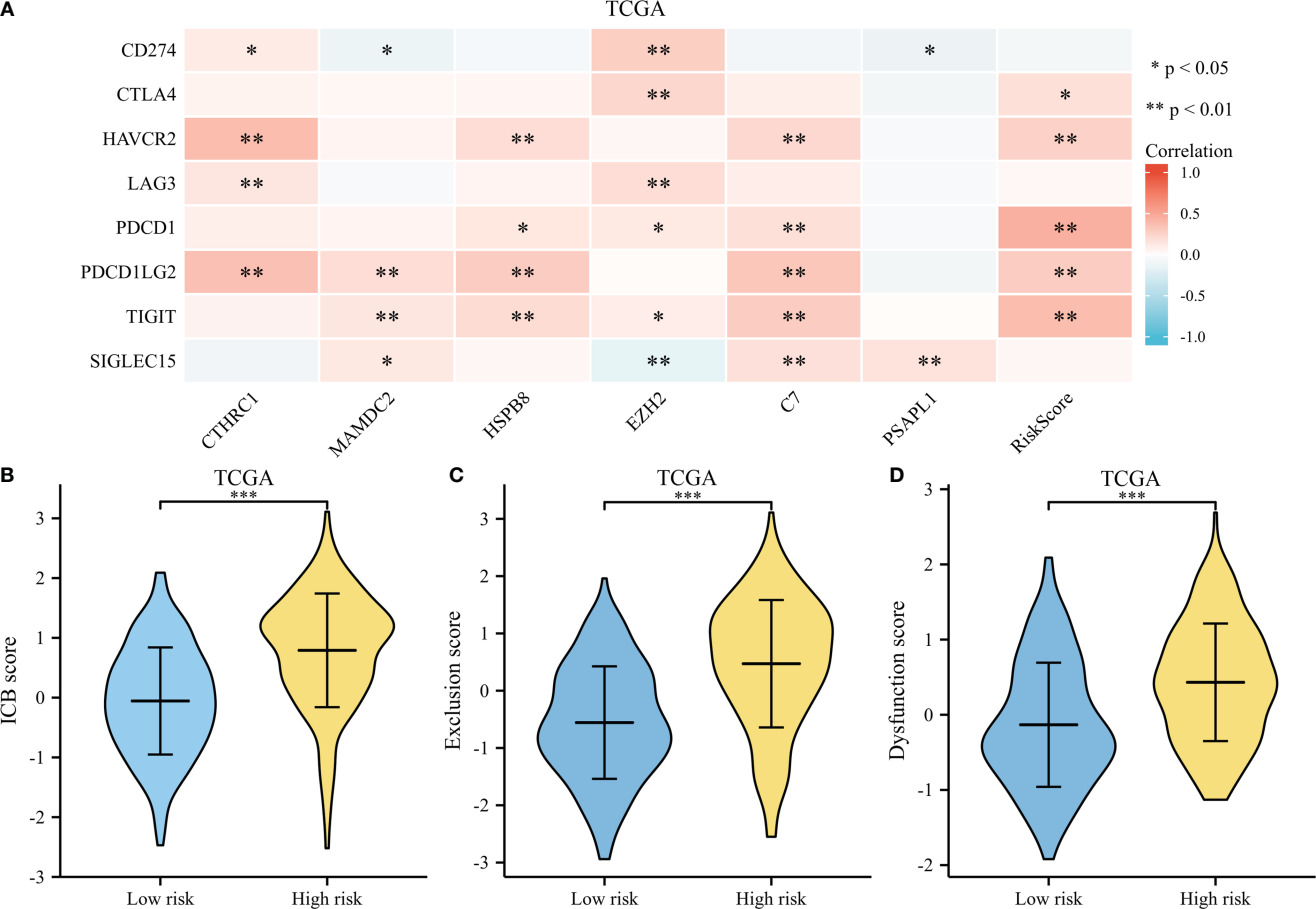
Immunotherapy response of GC. **(A)** A heatmap of immune-checkpoint expression and risk scores. **(B)** A violin plot comparing ICB scores for high-risk and low-risk. **(C)** A violin plot comparing Exclusion score for high-risk and low-risk. **(D)** A violin plot comparing Dysfunction score for high-risk and low-risk. *p < .05, **p < .01, and ***p < .001.

A heatmap illustrates survival-related gene expressions and immune-checkpoint-relevant genes (As depicted in **Figure 10A**). A notable association was observed across risk score and the expression of CTLA4, HAVCR2, PDCD1, PDCD1LG2, and TIGIT, indicating risk scores represent tumor-induced immunosuppression.

**Figure 11:**
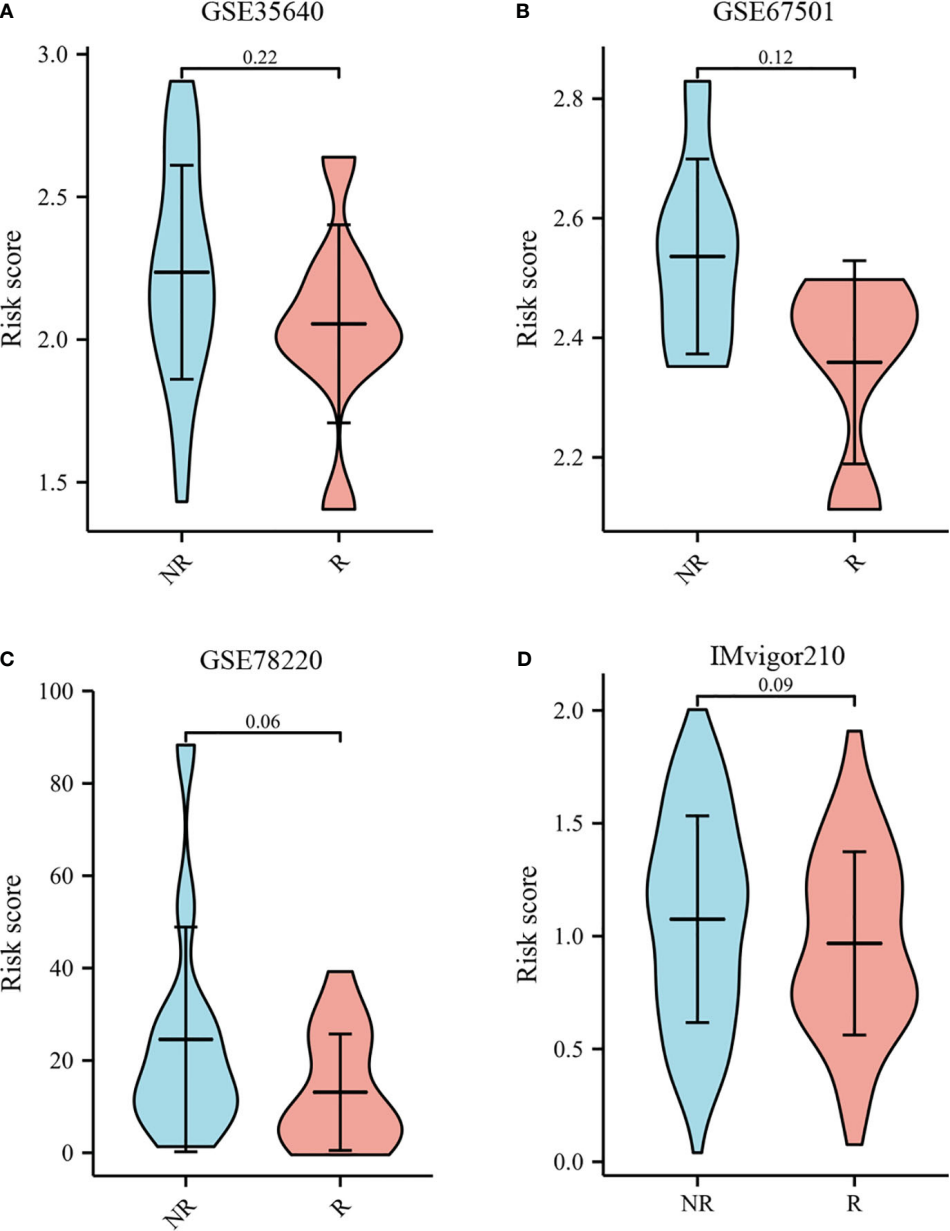
Immunotherapy response to immune checkpoints in the GSE35640, GSE67501, GSE78220, and IMvigor210 cohort.**(A)** The violin plot comparing responders and non-responders to immunotherapy in GSE35640. **(B)** The violin plot comparing responders and non-responders to immunotherapy in GSE67501. **(C)** The violin plot comparing responders and non-responders to immunotherapy in GSE78220. **(D)** The violin plot comparing responders and non-responders to immunotherapy in IMvigor210.

The TIDE score was used as a predictor of clinical outcome after immune checkpoint blockade. Risk groups differed significantly from low- and high-risk groups (depicted in **Figures 10B–D**). The TIDE scores, exclusion scores and dysfunction scores for low-risk were lower than high-risk group. In addition, we identified prognostic signature for immune checkpoint therapy response in the GSE78220, GSE35640, GSE67501, and IMvigor210 cohort. As shown in **Figures 11A–D**, there was a tendency for non-responders with higher risk scores than responders.

### 3.5 Nomogram validation and construction

**Figure 12:**
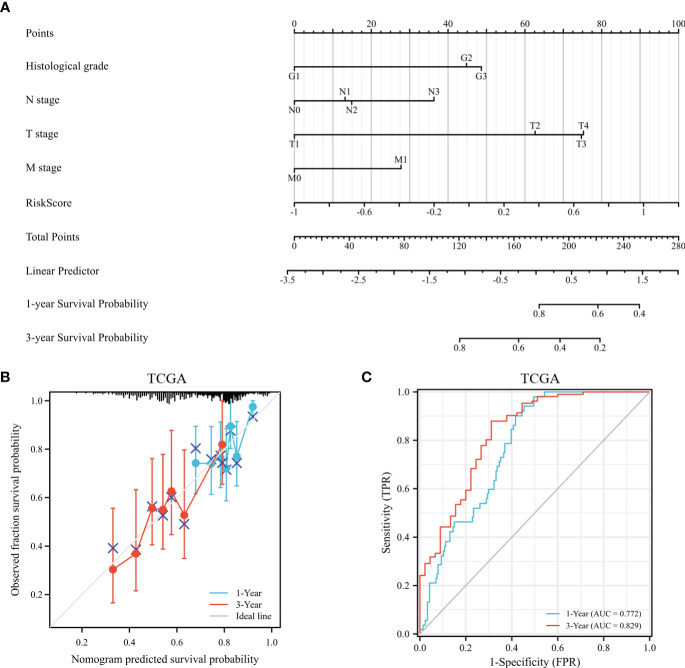
The assessment of a nomogram based on clinical characteristics and risk scores. **(A)** A nomogram based on the clinical parameters and risk model. **(B)** An analysis of the calibration curves for the TCGA dataset at 1-year survival and 3-year survival. **(C)** Time-dependent receiver operating curve that predicted overall survival.

According to survival-related gene signatures and clinical characteristics, a prognostic nomogram was developed. Univariate Cox regression analyses indicated that risk score, gender, age, clinical stage, and T stage are independent prognostic factors (depicted in **Supplementary Table 1**). Therefore, all of these factors were incorporated into a nomogram for the purpose of predicting the 1- and 3-year survival rates. Summing risk score and clinical parameters based on nomogram, the survival rate was calculated (**Figure 12A**). The nomogram calibration curves showed that 1-year and 3-year OS were in accordance (**Figure 12B**). Nomogram results indicate AUCs of 0.77 and 0.83 over 1- and 3-year periods (**Figure 12C**). Prognostic nomogram demonstrated greater accuracy in survival outcome predictions for GC patients.

## 4 Discussion

Over the past few years, the prognosis for GC patients has primarily been determined by clinical parameters, such as TNM stage, serum tumor biomarkers, and pathological types. Nevertheless, these factors are not useful for clinical decision-making due to their limited predictive efficacy. As a result, discovery of more efficacious biomarkers could make it easier for physicians to make individual treatment decisions. With the continuous evolution of sequencing technology, genomics could potentially play a significant role in identifying predictive biomarkers for various malignancies. There is little predictive value in a single gene for the outcomes of GC patients. In comparison to single gene models, multigene models are much more predictive than single gene models.

We studied DEGs between GC tissues and adjacent non-tumor tissues in GEO and TCGA datasets. There were 3211 DEGs in TCGA, 2371 DEGs in GSE54129, 627 DEGs in GSE66229, and 329 DEGs in GSE64951 selected as candidate genes and intersected with to obtain DEGs. In total, the 208 DEGs were further screened in TCGA cohort through univariate Cox regression analyses. Following this, LASSO regression was utilized to generate prognostic model of 6 DEGs. External validation showed favorable predictive efficacy. As a final step, nomogram was developed in this study, utilizing both clinical characteristics and a prognostic model.

The prognostic signature contains 6 biomarkers and categorizes GC patients into high- and low-risk group. Among 6 genes in the prognostic model, CTHRC1 was recognized as a novel gene involved in tissue remodeling and found to be overexpressed in carcinogenesis and metastasis of several solid cancers, as breast cancer (BC) (22) and non-small cell lung cancer (23). Gu (24) et al. investigated the relation of CTHRC1 expression and clinical features among GC patients. Patients with high CTHRC1 expression displayed poorer OS and disease-free survival (DFS) than low CTHRC1 expression patients. CTHRC1 has been reported to promoting cell migration and invasion through HIF-1α/CXCR4 signaling pathway in GC (25). Additionally, CTHRC1 overexpression induced tumor associated macrophage infiltration *via* AnxA1/FPR1 and GRN/TNFRSF1A signaling pathway, indicating CTHRC1 might be a promising predictive factor for immunotherapy (26).

MAMDC2 was differentially expressed between normal tissues and several solid tumors, including BC (27), head and neck squamous cell carcinoma (28), and GC (29). Meng (30) et al. identified MAMDC2 overexpression was significantly linked to poor DFS of BC. There have been controversial results reported in the literature (31). MAMDC2 was down-regulated in the BC cells. MAMDC2 Overexpression significantly suppressed proliferation and induced cell apoptosis *in vitro* and *in vivo*. There were contradictory reports on the involvement of MAMDC2 in tumor progression in the literature. In this study, high MAMDC2 expression patients experienced shorter OS than low MAMDC2 expression. MAMDC2 expression was inversely associated with CD4^+^ memory T cell, CD4^+^ effector memory T cell, CD8^+^ effector T cells, plasmacytoid dentritic cell, B cell plasma, CD4^+^ Th1 T cells, and gamma delta T cells. According to immune-checkpoint–relevant transcripts, MAMDC2 was strongly correlated to expression of PDCD1LG2, TIGIT, and SIGLEC15. Our findings suggest that MAMDC2 overexpression may contribute to immunological suppression in GC patients as well as a poor prognosis.

The stress-related protein HSPB8 was first discovered in human melanoma cells as a kinase of the H11 protein. HSPB8 Overexpression promoted proliferation, migration, and invasion (32, 33). HSPB8 suppressed mitochondrial impairment and aggravated proliferation and migration of A549 lung adenocarcinoma cells (34). In GC, HSPB8 expression was significantly linked to worse OS and recurrence-free survival (35). Furthermore, immune cell infiltrate analysis indicated B cells, CD4^+^T cells, and CD8^+^T cells were significantly different across high HSPB8 expression group and low HSPB8 expression group in bladder cancer (36).

The EZH2 methyltransferase is a core catalytic subunit of the polycomb repressive complex 2. The EZH2 protein mediates modifications in histone methylation that repress several tumor suppressor genes, as DKKI, CDH1, and DAB2IP (37). Zhao (38) et al. found EZH2 mediated EphB3 transcription through H3K27me3 modification, and inhibited tumor proliferation and metastasis by regulation E-cadherin and vimentin expression. Our findings indicate that high EZH2 expression patients had significantly longer OS than those with low EZH2 expression. The proportions of CD8^+^ effector memory T cells, CD8^+^ naive T cells, plasmacytoid dentritic cell, B cell plasma, CD4^+^ Th1 T cells, and gamma delta T cells were significantly elevated in low EZH2 expression patients, indicating EZH2 represented the activation of TME.

C7 was the final component of the complement cascade and essential for complement activation. Seol (39) et al. found C7 overexpression induced tumorsphere formation, and maintain stemness of liver cancer cells. However, several studies reported C7 displayed as a potential tumor suppressor and was related to tumor progression and prognosis for certain cancers (40, 41). Our findings suggested the high C7 expression patients had the trend to live a longer OS. Additionally, there was significant differences in the C7 expression levels across common lymphoid progenitor, plasmacytoid dendritic cell, CD4^+^ memory T cell, CD8^+^ naive T cell, gamma delta T cell, CD4^+^ Th1 T cell, and CD4^+^ Th2 T cell. The C7 expression had notable direct correlation with the expression of HAVCR2, PDCD1, PDCD1LG2, SIGLEC15, and TIGIT, indicating risk score was indicative of the extent of immunosuppression induced by tumors.

There was some literature about the oncogenic role of PSAPL1 in various solid cancers, including GC (42), BC (43), hepatocellular carcinoma (44). Our study indicated high expression PSAPL1 patients had a worse OS than those of low expression PSAPL1. According to immune scores and immune cell infiltration, there was no significant difference of stroma score, immune score, ESTIMATE score, and immune cell infiltration regard to PSAPL1 expression in GC patients. Further studies are required to elucidate PSAPL1 mechanisms in GC.

We studied interaction between risk models, immunoscores, and immune cell infiltrate based on six-gene signature. The high-risk group exhibited significantly elevated ESTIMATE score, immunescore, and stromal score relative to low-risk group. Utilizing abundance of tumor-infiltrating immune cells in each GC patient, risk scores were calculated. As a result, we identified that CD4^+^ memory T cells, CD8^+^ naive T cells, common lymphoid progenitor, mast cell, plasmacytoid dentritic cell, B cell plasma, regulatory T cells, gamma delta T cell, and B cell plasma relative fractions were significantly enriched in low-risk group. A notable correlation was observed between risk score and expression of CTLA4, HAVCR2, PDCD1, PDCD1LG2, and TIGIT, indicating risk scores represent tumor-induced immunosuppression. According to TIDE, the TIDE scores, exclusion scores and dysfunction scores for low-risk group were lower than those for high-risk group. TIDE algorithm and immune-checkpoint-relevant transcripts were consistent with xCell, suggesting immunosuppressive microenvironments were more common among high-risk GC patients.

This study may have major implications for patients with GC as far as prognosis and treatment are concerned. To help with clinical practice and risk classification, we offered a new signature. Low-risk individuals had longer life periods and were more likely to benefit from ICIs. In addition, we found a number of crucial genes that could serve as GC treatment targets. Several previous studies (45–47) constructed mRNA prognosis signatures in GC patients. The AUC values of these studies ranged from 0.54 to 0.62, which were inferior to the current study. We examined prognostic model significance for other solid cancer in addition to researching immunotherapy response and prognosis among GC patients.

Several limitations were identified in our study. Firstly, data on mRNA expression and clinical information were downloaded from an open-source database. The findings of these studies have not yet been confirmed in clinical trials. The environment, genetics, and epigenetics are also factors influencing GC. Further molecular biological studies are necessary to verify involvement of the 6 DEGs in GC progression. Finally, due to TCGA data unavailability, the risk model was unable to provide the predictive value compared to a number of widely used predictors, such as pathological grade and treatment approach.

In conclusion, we discovered a 6 gene signature to forecast GC patients’ OS. This risk signature proves to be a valuable clinical predictive tool for guiding clinical practice. Moreover, the profile revealed discernible variations in immune cell infiltration levels and immunotherapy response among low- and high-risk groups. This prognostic model thus offers a precise and impartial basis for directing unique therapy choices for GC.

## Data availability statement

Publicly available datasets were analyzed in this study. This data can be found here: TCGA repository (https://portal.gdc.cancer.gov/), GEO database (https://www.ncbi.nlm.nih.gov/geo/query/acc.cgi), and IMvigor210 (http://research-pub.gene.com/IMvigor210CoreBiologies/).

## Author contributions

QW and BZ designed the study. HW, HF and WG collected the data. MH, TG and HL performed the research. QW wrote the paper. XJ, MX and YD analyzed the data. All authors contributed to the article and approved the submitted version.
