# Supplementary material for: Identification of a six-gene signature to predict survival and immunotherapy effectiveness of gastric cancer
Source: Front Oncol. 2023 Jun 19;13:1210994. doi: 10.3389/fonc.2023.1210994 (PMC10316024; doi:10.3389/fonc.2023.1210994)
Supplement: Supplementary file 1 [file Table_1.docx]

Supplementary Table 1. The univariate Cox regression analyses of the risk model and overall survival of GC patients in TCGA cohort

| Characteristics | Univariate analysis | |
| --- | --- | --- |
|  | Hazard ratio (95% CI) | P value |
| gender | 1.022 (1.005 - 1.039) | 0.009 |
| MALE | Reference |  |
| FEMALE | 0.771 (0.541-1.101) | 0.152 |
| Clinical stage` |  |  |
| I | Reference |  |
| II | 1.553 (0.783-3.082) | 0.208 |
| III | 2.385 (1.258-4.522) | 0.008 |
| IV | 3.827 (1.855-7.898) | <0.001 |
| Histological grade |  |  |
| G1 | Reference |  |
| G2 | 1.647 (0.400-6.783) | 0.490 |
| G3 | 2.150 (0.529-8.739) | 0.285 |
| N stage |  |  |
| N0 | Reference |  |
| N1 | 1.629 (1.001-2.649) | 0.049 |
| N2 | 1.656 (0.980-2.798) | 0.060 |
| N3 | 2.647 (1.627-4.307) | <0.001 |
| T stage |  |  |
| T1 | Reference |  |
| T2 | 6.729 (0.914-49.550) | 0.061 |
| T3 | 9.428 (1.309-67.896) | 0.026 |
| T4 | 9.652 (1.325-70.285) | 0.025 |
| M stage |  |  |
| M0 | Reference |  |
| M1 | 2.100 (1.184-3.724) | 0.011 |
| RiskScore | 2.718 (1.736-4.255) | <0.001 |
